# The interoperability of crystallographic data and databases

**DOI:** 10.1107/S2052252523010424

**Published:** 2024-01-01

**Authors:** Alice Brink, Ian Bruno, John R. Helliwell, Brian McMahon

**Affiliations:** a Chemistry Department, University of the Free State, Nelson Mandela Drive, Bloemfontein, South Africa; b Cambridge Crystallographic Data Centre, 12 Union Road, Cambridge CB2 1EZ, United Kingdom; c Department of Chemistry, University of Manchester, Oxford Road, Manchester, United Kingdom; d International Union of Crystallography, 5 Abbey Square, Chester CH1 2HU, United Kingdom; Formby, Liverpool, United Kingdom

**Keywords:** interoperability, data, databases, interdisciplinarity, cross-domain integration, CODATA

## Abstract

Interoperability of crystallographic data with other disciplines is essential for the smooth and rapid progress of structure-based science in the computer age. Within crystallography and closely related subject areas, there is already a high level of conformance to the generally accepted FAIR principles (that data be findable, accessible, interoperable and reusable) through the adoption of common information exchange protocols by databases, publishers, instrument vendors, experimental facilities and software authors. Driven by the success within these domains, the IUCr has worked closely with CODATA (the Committee on Data of the International Science Council) to help develop the latter’s commitment to cross-domain integration of discipline-specific data. The IUCr has, in particular, emphasized the need for standards relating to data quality and completeness as an adjunct to the FAIR data landscape. This can ensure definitive reusable data, which in turn can aid interoperability across domains. A microsymposium at the IUCr 2023 Congress provided an up-to-date survey of data interoperability within and outside of crystallography, expounded using a broad range of examples.

## Abstract 2

Interoperability of scientific data within crystallography has been achieved to a high level by the adoption of standard exchange formats and protocols. Extending such interoperability across other disciplines is a goal of the International Science Council Committee CODATA, to which the IUCr has been a leading contributor. Our article combines a description of the importance of interoperability for addressing grand challenges with a desire to stimulate the crystallographic community to continue exploring this topic.

## 1. Introduction

Interoperability in science is a very broad activity across the disciplines and within which the IUCr has been a leading contributor notably to the International Science Council’s Committee on Data, ‘CODATA’. In addition, within crystallography itself interoperability is still developing. Below we provide separate sections which cover these two aspects. We commence first with a section on broadly agreed foundational concepts.

### 1.1. Foundational concepts

The FAIR principles (Wilkinson *et al.*, 2016 ▸) that data must be findable, accessible, interoperable and reusable have found a very wide traction in the scientific community as they promote the optimized reuse of data.

There has been detailed consideration of what makes a dataset FAIR (Wilkinson *et al.*, 2018 ▸; FAIR Data Maturity Working Group, 2020 ▸). Enablers for implementing FAIR include adoption of persistent and standard identifiers, describing datasets with rich metadata based on community standards, use of standard access protocols, and ensuring that provenance and reuse conditions are clear. Specifically for interoperability, the use of formal languages and consistent vocabularies across disciplines for knowledge representation is considered key along with inclusion of qualified references to other relevant data. That said, vocabularies utilized even in closely allied subjects such as chemistry and biochemistry do not necessarily have identical meanings (Brink & Helliwell, 2019 ▸).

Crucially, the FAIR data principles target discovery and reuse of data by machines and not just individuals where human interpretation comes into play. There is an increased reliance on computational support to process and identify information as a result of the increase in volume, complexity and creation speed of data. Interoperability is compromised if the data are misleading. Hence, the IUCr published a recommendation (Hackert *et al.*, 2016 ▸) that scientific data should be:

*Complete*, *i.e.* all data collected for a particular purpose should be available for subsequent re-use.

*Precise*, the meaning of each datum is appropriately defined, processing parameters are fully specified and quantified, statistical uncertainties have been evaluated and declared.

This recommendation was contained in a formal response from the IUCr to an influential report published in 2015 entitled ‘Open Data in a Big Data World’ (Science International, 2015 ▸). In a general way this landmark document described ‘the values of open data in the emerging scientific culture of big data’. The IUCr acknowledged the importance of this accord and endorsed the analysis of the value of open data and the ‘Principles of Open Data’ set out in the document. Because the specific values, significance and implementation of open data principles vary in detail between disciplines, the IUCr considered it useful to contribute a detailed response, as a case study of best practice emerging in one field, that of crystallography. It emphasized that in implementing data quality standards there is a diverse ecosystem of crystallographic databases, data repositories, experimental facilities and publishers, some sustained through subscription-based access but at no charge to the author/depositor, and without public funding. These approaches to sustainability and quality assurance serve crystallography well. We emphasize here that ensuring data quality and standards serves interoperability well too.

Note that FAIR data need not necessarily be available without constraint for everyone namely ‘open’, *i.e.* data should be as open as possible but as closed as necessary for reasons of safety or security. A classic case of ‘as closed as necessary’ is that of a database of the locations of rare birds or rhino where access to such a dataset should be strictly controlled to prevent the abuse of knowledge.

Recent discussions within crystallographic communities have focused on the merits and practicability of storing experimental raw data (*e.g.* X-ray diffraction images) in open repositories. As these represent essentially loss-free information about the crystal structure being probed, they would certainly satisfy the completeness requirement which is identified above. The size of such datasets does raise technical and economic challenges to making these routinely available. The IUCr Committee on Data and IUCr journals are actively working to establish circumstances where such data are most usefully retained, and to allow commentary on datasets with particularly interesting features that are not fully described in a derived structural model, but are made accessible under the FAIR principles for additional scrutiny (Kroon-Batenburg *et al.*, 2022 ▸).

### 1.2. Across the scientific disciplines activities and IUCr’s participation

Achieving interoperability of datasets across disciplines is a significant challenge. For example, CODATA has been working for at least five years on ‘cross-domain integration to facilitate a more effective tackling of challenges such as disaster risk reduction’ (https://codata.org/initiatives/decadal-programme2/ddi-cross-domain-integration/). At that level where might crystallography contribute? In recent times, a disaster that happened was the COVID-19 outbreak. The structural sciences community comprising crystallographers, diffractionists, scatterers, microscopists and spectroscopists came together to characterize the 3D structures and properties of this virus and its components.

A key step on behalf of crystallographic databases within the CODATA and Research Data Alliance (2023 ▸) communities describing the inter-disciplinary relevance of crystallography was made at International Data Week 2016 in Denver where we (JRH and BM) organized a session on Crystallographic Databases. This session featured the largest databases, and from which an article was published by the speakers in CODATA’s *Data Science Journal* (Bruno *et al.*, 2017 ▸). This article covered a very broad range of databases, the larger ones, and the smaller more specialized ones. The scientific diversity of these databases highlights the important role crystallography plays in many different sciences (and, as a consequence, the need to make their contents easily findable and accessible to researchers in other domains – and, of course, easily usable).

**Figure 1:**
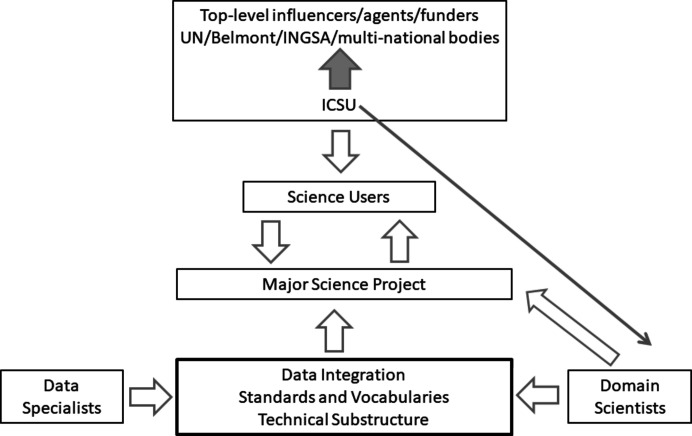
‘Hierarchy of engagement’ of stakeholders working on data interoperability (after presentations at the CODATA workshops discussed in the text). The box with a heavy outline indicates how crystallography has well defined standards and vocabularies (mostly within the CIF framework, see main text), and significant technical substructure whereby the crystallographic databases offer access through APIs and, usually, SQL or other common database standard query languages. To extend this, and thereby facilitate data integration with other domains, we suggest well characterized data formats and well characterized metadata will be needed as well as inter-domain protocols such as format conversions, metadata concordances, versioning conventions and access control mechanisms.

CODATA prepared its position on ‘data integration’ and an accompanying roadmap very carefully by organizing two workshops, one in Paris and one in London in 2017. The participants were drawn from members of the International Council of Scientific Unions (ICSU), the representatives of the International Social Science Council and standards bodies such as the World Wide Web Consortium (W3C, https://www.w3.org/). The purpose of these activities was to share details of our data and information activities, agree on good practice, and seek consensus about how Unions and disciplinary groups can best work together in establishing a global network of scientific data that is FAIR. The major output of the meeting was a Roadmap of Priorities for inter-Union cooperation which led to the WorldFAIR project (see below and https://codata.org/initiatives/decadal-programme2/worldfair/). These activities show a connection between WorldFAIR and CODATA’s contribution to the International Science Council (ISC) Action Plan Project 2.1, Making Data Work For Cross-Domain Grand Challenges (https://council.science/actionplan/making-data-work-for-grand-challenges/). A sum­mary of the way in which interoperability of data might be organized is shown in Fig. 1 ▸.

The CODATA Workshop talks encompassed broad themes and Scientific Unions’ short presentation summaries of good practice. Aside from the IUCr, the Unions who presented were the International Union of Pure and Applied Chemistry (IUPAC), the International Union of Geodesy and Geophysics (IUGG), and the International Union of Geological Sciences (IUGS). The IUCr’s talk (by JRH) emphasized the vital role of data characterization and quality as exemplified by the Crystallographic Information Framework and how this is supported by COMCIFS (https://www.iucr.org/resources/cif/comcifs), *checkCIF* (or wwPDB validation reports), and human review by editors and referees of the data underpinning articles (structure factors and coordinates) published in IUCr Journals (Hall & McMahon, 2016 ▸). A useful account of the relevance of CIF to initiatives in developing shared metadata standards across a broader spectrum of materials science applications is given by Ghiringhelli *et al.* (2023 ▸). Within this workshop report, two further possible definitions are introduced for the ‘R’ in FAIR [R = reusability in Wilkinson *et al.* (2016 ▸)], namely ‘repurposable’ and ‘recyclable’.

Within the Paris Workshop an especially impressive approach to data interoperability itself was from the Ocean Data Interoperability Platform. This nicely documented the nature of interdisciplinarity and the diverse data required, and then explicitly the need for interoperability. Quoting from their website (http://www.odip.org/):

The Ocean Data Interoperability Platform (ODIP) contributes to the removal of barriers hindering the effective sharing of data across scientific domains and international boundaries. ODIP includes all the major organizations engaged in ocean data management in the EU, US and Australia.

Schaap (2017 ▸, slide 2) claims that ODIP overcomes barriers by being a community-led initiative, exploring common standards and interoperability solutions for improving the exchange between regional infrastructures and towards global infrastructures such as the Global Earth Observation System of Systems (GEOSS) and UNESCO’s International Oceanographic Data and Information Exchange programme (IOC/IODE). To make a comparison with IUCr here, stakeholder consensus is important. We highlight that the crystallography community has a high degree of participation in IUCr’s standards-setting efforts.

### 1.3. Specific developments in reaching definitive protein crystal structures as they are accessed most often by non-crystallographers

There are situations that arise where datasets require post-publication peer review [an early account of reasons for this was given by Terwilliger (2012 ▸)] and so methods for versioning must be defined and the processes for their updating agreed by researchers, both those who originally measured and analysed the data, but also those who in their reuse of data may make an improved interpretation. A common category of new and improved interpretation involves new or improved software for enhanced calculations with the original data. A very important point is that versioning of software is necessary to understand a given processed and/or derived dataset at any one time that is obtained by that software. That multiple versions can confuse interoperability has been highlighted by CODATA Global Open Science Cloud Case Study 5 ‘Open reproducible raw diffraction data for access in pandemics’ [https://codata.org/initiatives/decadal-programme2/global-open-science-cloud/case-studies/diffraction-data/ and Hell­iwell *et al.* (2023 ▸)]. This case study offers a procedure for ‘definitive reusability’ via the PDB as a single place of contact for protein structures and which can thereby facilitate a better interoperability than multiple websites, each claiming to offer a better protein structure, derived from a single measured diffraction dataset by an original experimental team. This sets a challenge, and a framework how to solve a difficulty within a scientific discipline where 99% of PDB data consumers are not experts in structural biology (Burley *et al.*, 2022 ▸). The impacts of the CSD are also broad [see table 4 of Willett *et al.* (2020 ▸)] again emphasizing the importance of interoperability between disciplines.

Within this versioning protocol the PDB archive allows access to previous datasets which is important (1) for full transparency and auditing, and (2) to understand the contemporaneous reasons for drawing the conclusions published from the original dataset.

In facing a societal challenge like HIV, crystallography brought together biological and chemical three-dimensional structure results to identify treatments for HIV (see https://pdb101.rcsb.org/motm/6 for a general description). The biological and chemical crystal structure databases provided the durable archiving of precise and accurate data for new compound design and thus the scientific process involved firmly rested on these across-the-disciplines data.

## 2. Conceptualization of the Microsymposium at IUCr 2023

At the IUCr Congress and General Assembly held in Melbourne, Australia, the session entitled ‘The Interoperability of Crystallographic Data and Databases’ explored the way crystallographic data can be appropriately applied across disciplines to enable the advancement of science. Interdisciplinary research requires a common crystallographic language, an ability to unambiguously understand the crystallographic information, and data interoperability – the property that allows for the reliable sharing of resources or data between different systems. In an age of data-driven science, these attributes are required for processing of data by machines as well as people.

The session showcased initiatives relating to databases, software and research which promote the sharing of crystallographic knowledge and encourage the use of crystallographic data across disciplines and ages. In particular it focused on how data from different disciplines may be accessed, manipulated and cross-utilized as well as the challenges hindering the interoperability of currently available databases. The benefits, practical solutions and learning initiatives enabled by data interoperability were also described.

The session fostered awareness of data activities within various disciplines that are improving interoperability across domains and databases. There were six presentations which focused on access, use and reuse of data across disciplines and highlighted scientific case studies that demonstrated the benefits and challenges of using data from chemistry, biology, materials, computational studies and crystallography (chemical, macromolecular or powder) in cross-disciplinary research to address research questions relevant to societal challenges.

These presentations demonstrated the high level of interoperability that already exists among stakeholders in the structural sciences, but also emphasized the need to press forward with initiatives to bridge new cross-disciplinary requirements. As recently as October 2023, the Protein Data Bank announced that electron microscopy metadata would be distributed from the Electron Microscopy Data Bank in the common PDBx/mmCIF format to improve interoperability between those two archives (wwPDB, 2023 ▸).

## 3. Proceedings and transactions in the Microsymposium

The talks presented are listed in Table 1 ▸. The diversity of these talks showed that interoperability covers both a wide potential audience (in the sense that databases are used by chemists, materials scientists, biologists, lawyers *etc.*) and the narrower user community within laboratories that must ensure appropriate handling of the chemical, biological *etc.* ramifications of a crystallographic study.

Relating to the CODATA overview, the contributions by Suzanna Ward of the CCDC and by Soorya Kabekkodu of the ICDD are very pertinent:

Suzanna Ward (Lightfoot *et al.*, 2023 ▸) explored the role that the CCDC plays in enabling reliable sharing of data between disciplines and efforts to adopt best practices for data management that enable researchers to get the most from crystal structure data. One important enabler is generation of a machine-readable representation of the chemical substance studied by a diffraction experiment. This is rarely provided as part of a publication of a crystal structure dataset and without it the extent to which a crystal structure is interoperable across disciplines is severely limited. Interoperability is enhanced if the chemical representation can be mapped onto the experimentally determined 3D structure. This requires treatment of disorder and identification of an optimal monomer unit for polymeric structures. Automated procedures that aim to enrich crystal structures in this way have been established (Bruno *et al.*, 2011 ▸), but curation by human experts remains necessary to ensure the results are reliable.

A reliable chemical representation is an important precursor for linking chemical crystallographic data to data in other chemical and biological data resources. A challenge here is that different resources may use different conventions to represent bond types in a chemical structure. The International Chemical Identifier (InChI) (Heller *et al.*, 2013 ▸) offers a solution to this challenge. The InChI provides a canonical normalized identifier for a chemical structure that is common for different but equivalent chemical representations. It is used by the CCDC to connect crystal structure data to chemical and biological data resources including those maintained by the EBI (BioChemGRAPH, https://gtr.ukri.org/projects?ref=BB/T019778/1). Interoperability is further supported through the provision of Application Programming Interfaces (APIs) that enable researchers to embed access to crystallographic data in wider workflows and integration into computational chemistry and structural biology software solutions (https://www.ccdc.cam.ac.uk/solutions/software/csd-python/).

BioChemGRAPH, mentioned above, is intended to interoperate within the framework of PDBe-KB, a knowledge base project, under the aegis of the Protein Data Bank in Europe that provides functional annotations for protein structures with input from over twenty resources across the life sciences community (PDBe-KB consortium, 2021 ▸). The relationship between the various ontologies involved in this cross-domain activity is demonstrated visually in the PDBe Graph Database Schema Explorer (https://www.ebi.ac.uk/pdbe/pdbe-kb/schema/).

Underpinning much of this is the fact that crystallographic data are initially published using a common knowledge representation language, *i.e.* CIF. CCDC data-deposition workflows are centred on CIF and aim to make it as easy as possible for researchers to provide data, information and knowledge in a form that can be made available through wider interoperability frameworks. An area where these frameworks currently fall short is enabling access to physical and chemical properties relevant to a crystal structure. Even if links between crystal structure databases and other information resources can be established there is often insufficient context to know if a property is relevant and can be reused. Such properties are critical for training predictive machine-learning models, some of which also require negative data (*e.g.* an indication of crystallization attempts that failed as well as those that succeeded). In essence, successful interoperability relies on rich reporting of data in the first instance and thus on the implementation of other aspects of FAIR, reusability in particular.

Soorya Kabekkodu (Kabekkodu & Blanton, 2023 ▸) explored how the ICDD PDF is a curated database with every entry quality marked using a combination of computer and human editorial review. We would observe that a key role of powder diffraction data is in resolving legal disputes between different pharmaceutical companies as to whether new polymorphs have been discovered, and so quality marks are clearly a pivotal aspect. For examples of such courtroom cases, see Bernstein (2020 ▸, chapter 10). These emphasize the wider need for such data to be interoperable, to be understandable by legal teams and the presiding judge in any given courtroom case. Quality indicators also allow subsets of the hits returned for a particular query to be selected. This approach is used as well in other databases for instance the CSD and wwPDB with the IUCr *checkCIF* report and PDB validation report.

Within individual laboratories the talks from both Newcastle and Durham Universities emphasized the day-to-day practical details of managing both crystallizations and crystal structure determinations in conjunction with the relevant experimental metadata in a convenient and structured manner. This approach is a necessary consideration for principal investigators wishing to establish sustainable research incorporating the span of several student generations passing through a research laboratory. For the crystal structure determinations service the other aspect of interoperability for such a system is that it also enables sharing of information and data between other analytical services that synthetic chemists use, for example solid-state NMR, PXRD, IR *etc*. This is a long-standing part of the culture of chemistry to have an integrated structural chemistry culture (*e.g.* Ebsworth, 1991 ▸).

## 4. The future of interoperability

The future of interoperability across different disciplines may rest on the formal development of an information-exchange protocol stack, where standards are created to convert between specific disciplinary formats and ontologies (McMahon, 2017 ▸). However, recent advances with large-language models (such as ChatGPT) suggest it may also involve machine learning and artificial intelligence in some form. Brian McMahon described new approaches (*e.g.* Özer *et al.*, 2022 ▸) using machine learning trained on standardized datasets that help to address the challenge of making scientific connections across different disciplines with appropriate metadata catalogues.

Machine-to-machine ‘conversations’, rather than human-to-human or human-to-machine conversations, have aroused anxieties at the possible confusions in the digital representation of units. So, CODATA has again been very active in setting up a Task Group on the Digital Representation of Units of Measurement (DRUM): https://codata.org/initiatives/task-groups/drum/. The objective of this CODATA Task Group is to work with the International Science Unions to raise awareness of, educate and enable their communities in the understanding and implementation of digital unit representation.

Within the crystallography discipline we note that Brink & Helliwell (2019 ▸) raised several interoperability challenges between the chemical and macromolecular formats experienced during their Re/Tc organometallic structural crystallographic research with application for medical imaging. Work to address the suggestions made has commenced.

Within the IUCr 75th anniversary session at the IUCr Melbourne Congress, Gautam Desiraju spoke as a recent past President of the IUCr and described a possible future vision of the IUCr, the subject of an opening day Workshop of the Congress. He described a switch of emphasis from three-dimensional structures to organizing compounds and their crystal structures by ‘properties’. This vision connected well with the presentation in our session by D Matulis who described, within his Protein–Ligand Binding Database, (PLBD) the interoperability of thermodynamic and kinetic data of protein interaction with small-molecule compounds within the 127 X-ray crystal structures of six CA isozyme complexes with ligands (Lingė *et al.*, 2023 ▸).

## 5. Concluding remarks

We conclude this article by returning to the two CODATA Data Integration Workshops which defined the overall objectives to harness quantitative science (*i.e.* data) to help address societal challenges such as disaster risk reduction, smart cities, clean air, agriculture and population, sea-level rise, security, preservation of heritage, wearable technologies, biodiversity, poverty and migration, the future of humankind and of the Earth. We can define these objectives as ‘grand challenges’. Aspects of some of these challenges are being tackled within the CODATA/RDA WorldFAIR project funded by the EU. This comprises a set of disciplinary and cross-disciplinary case studies that aim in particular to improve digital research objects, including data. It is anticipated that these case studies will inform a drive towards guidelines for domain-agnostic standards that truly work across disciplines and institutions providing a Cross-Domain Interoperability Framework (CDIF; https://worldfair-project.eu/cross-domain-interoperability-framework/).

It may well not be possible for crystallography to contribute to each societal challenge in a substantial manner, but the community can certainly be on hand to witness and join discussions where appropriate. A particular example where crystallography definitely helped was in the CODATA VAMAS Nanomaterials definition document (https://codata.org/initiatives/previous-codata-working-groups/nanomaterials/). At its core was a need for structure and properties characterization as the foundation for defining situations where safety might be a concern. Thus, claims can be avoided, or side-stepped, that all nanoparticles are unsafe as were exemplified by such as Michael Crichton’s alarmist novel ‘Micro’ [https://en.wikipedia.org/wiki/Micro_(novel)].

It is within these very broad contexts that CODATA stimulates all of us as scientists to reflect deeply on data interoperability. We hope that this article describing the IUCr 2023 session on interoperability of crystallographic data and databases may stimulate future microsymposia to be organized on these important themes.

## Figures and Tables

**Table 1:** The presented talks at the 2023 IUCr Microsymposium Note: these Congress abstracts are cited in the references list below and are a supplement to *Acta Cryst. A* accessible in the IUCr Journals.

*Applications of metadata collection and analysis to parallel crystallization and the ENaCt technique* by Smith, Probert & Hall (2023 ▸), Newcastle University; presented by Tom Smith. See also Tyler *et al.* (2020 ▸) for details of Encapsulated Nanodroplet Crystallization (ENaCt).
*DIMAS: a web-based service crystallography submission and data management system* by Blundell & Dolomanov (2023 ▸), Durham University; presented by Toby Blundell.
*Protein–Ligand Binding Database (PLBD) of Crystal Structures and Intrinsic Thermodynamic Parameters* presented by Matulis (2023 ▸), Vilnius University, on behalf of 22 other coauthors.
*Database interoperability: a powder diffraction perspective* by Kabekkodu & Blanton (2023 ▸), ICDD; presented by Soorya Kabekkodu.
*Interoperability of Databases as viewed by the Publisher* presented by McMahon (2023 ▸), IUCr.
*The Cambridge Structural Database: a multidisciplinary resource* by Ward & Lightfoot (Lightfoot *et al.*, 2023 ▸), CCDC; presented by Suzanna Ward.

## References

[bb1] Bernstein, J. (2020). *Polymorphism in Molecular Crystals*, 2nd Ed. p. 608. Oxford University Press.

[bb3] Blundell, T. & Dolomanov, O. (2023). *Acta Cryst.* A**79**. In the Press.

[bb4] Brink, A. & Helliwell, J. R. (2019). *IUCrJ*, **6**, 788–793. 10.1107/S2052252519010972 PMC6760442 31576212

[bb5] Bruno, I., Gražulis, S., Helliwell, J. R., Kabekkodu, S. N., McMahon, B. & Westbrook, J. (2017). *Data Sci. J.* **16**, 38.

[bb6] Bruno, I. J., Shields, G. P. & Taylor, R. (2011). *Acta Cryst.* B**67**, 333–349. 10.1107/S0108768111024608 PMC3143025 21775812

[bb7] Burley, S. K., Berman, H. M., Duarte, J. M., Feng, Z., Flatt, J. W., Hudson, B. P., Lowe, R., Peisach, E., Piehl, D. W., Rose, Y., Sali, A., Sekharan, M., Shao, C., Vallat, B., Voigt, M., Westbrook, J. D., Young, J. Y. & Zardecki, C. (2022). *Biomolecules*, **12**, 1425. 10.3390/biom12101425 PMC9599165 36291635

[bb8] Ebsworth, E. A. V. (1991). *Structural Methods in Inorganic Chemistry.* 2nd edition. Blackwell Scientific Publicatons.

[bb9] FAIR Data Maturity Model Working Group. (2020). *FAIR Data Maturity Model. Specification and Guidelines (1.0)*. https://doi.org/10.15497/rda00050.

[bb10] Ghiringhelli, L. M., Baldauf, C., Bereau, T., Brockhauser, S., Carbogno, C., Chamanara, J., Cozzini, S., Curtarolo, S., Draxl, C., Dwaraknath, S., Fekete, Kermode, J., Koch, C. T., Kühbach, M., Ladines, A. N., Lambrix, P., Himmer, M., Levchenko, S. V., Oliveira, M., Michalchuk, A., Miller, R. E., Onat, B., Pavone, P., Pizzi, G., Regler, B., Rignanese, G., Schaarschmidt, J., Scheidgen, M., Schneidewind, A., Sheveleva, T., Su, C., Usvyat, D., Valsson, O., Wöll, C. & Scheffler, M. (2023). *Sci Data*, **10**, 626. 10.1038/s41597-023-02501-8 PMC10502089 37709811

[bb11] Hackert, M. L., van Meervelt, L., Helliwell, J. R. & McMahon, B. (2016). *Open Data in a Big Data World: a Position Paper for Crystallography.* https://www.iucr.org/iucr/open-data.

[bb12] Hall, S. R. & McMahon, B. (2016). *Data Sci. J.* **15**, 3.

[bb13] Heller, S., McNaught, A., Stein, S., Tchekhovskoi, D. & Pletnev, I. (2013). *J. Cheminform*, **5**, 7. 10.1186/1758-2946-5-7 PMC3599061 23343401

[bb14] Helliwell, J., Kurisu, G. & Kroon-Batenburg, L. (2023). *Acta Cryst.* A**79**. In the press.

[bb15] Kabekkodu, S. & Blanton, T. (2023). *Acta Cryst.* A**79**. In the Press.

[bb16] Kroon-Batenburg, L. M. J., Helliwell, J. R. & Hester, J. R. (2022). *IUCrData*, **7**, x220821. 10.1107/S2414314622008215 PMC9635430 36337453

[bb17] Lightfoot, M. P., Bruno, I. J., Johnson, N. T., Olatunji-Ojo, Y. & Ward, S. C. (2023). *Acta Cryst.* A**79**, a98.

[bb18] Lingė, D., Gedgaudas, M., Merkys, A., Petrauskas, V., Vaitkus, A., Grybauskas, A., Paketurytė, V., Zubrienė, A., Zakšauskas, A., Mickevičiūtė, A., Smirnovienė, J., Baranauskienė, L., Čapkauskaitė, E., Dudutienė, V., Urniežius, E., Konovalovas, A., Kazlauskas, E., Shubin, K., Schiöth, H. B., Chen, W., Ladbury, J. E., Gražulis, S. & Matulis, D. (2023). *Database*, **2023**, baad040. 10.1093/database/baad040 PMC10250011 37290059

[bb20] Matulis, D. (2023). *Acta Cryst.* A**79**. In the Press.

[bb21] Matulis, D. (2019). Editor. *Carbonic Anhydrase as Drug Target: Thermodynamics and Structure of Inhibitor Binding*. Springer Nature.

[bb22] McMahon, B. (2017). *CODATA and (Meta)data Characterisation in the Wider World*. Presentation at DDDWG Workshop on Metadata for raw data from X-ray diffraction and other structural techniques. https://www.iucr.org/__data/assets/pdf_file/0012/114123/15_McMahon.pdf

[bb23] McMahon, B. (2023). *Acta Cryst.* A**79**. In the Press.

[bb24] Özer, B., Karlsen, M. A., Thatcher, Z., Lan, L., McMahon, B., Strickland, P. R., Westrip, S. P., Sang, K. S., Billing, D. G., Ravnsbaek, D. B. & Billinge, S. J. L. (2022). *Acta Cryst.* A**78**, 386–394. 10.1107/S2053273322007483 PMC9434602 36047395

[bb25] PDBe-KB consortium (2021). *Nucl. Acids Res.* **50**, D534–D542. 10.1093/nar/gkab988 PMC8728252 34755867

[bb26] Research Data Alliance (2023). Metadata Standards Catalog. https://rdamsc.bath.ac.uk/.

[bb27] Schaap, D. M. A. (2017). *Ocean Data Interoperability Platform (ODIP) I and II.* http://www.odip.org/media/odip/org/documents/odip-agu2017-schaap-dec2017.pdf.

[bb28] Science International (2015). *Open Data in a Big Data World.* Paris: International Council for Science (ICSU), International Social Science Council (ISSC), The World Academy of Sciences (TWAS), InterAcademy Partnership (IAP).

[bb29] Smith, T., Probert, M. & Hall, M. (2023). *Acta Cryst.* A**79**. In the Press.

[bb30] Terwilliger, T. C. (2012). *ICSTI Insights: The Living Publication*. http://doi.org/10.2172/1043003.

[bb31] Tyler, A. R., Ragbirsingh, R., McMonagle, C. J., Waddell, P. G., Heaps, S. E., Steed, J. W., Thaw, P., Hall, M. J. & Probert, M. R. (2020). *Chem*, **6**, 1755–1765. 10.1016/j.chempr.2020.04.009 PMC7357602 32685768

[bb32] Wilkinson, M. D., Sansone, S., Schultes, E., Doorn, P., Bonino da Silva Santos, L. O. & Dumontier, M. (2018). *Sci. Data*, **5**, 180118. 10.1038/sdata.2018.118 PMC6018520 29944145

[bb33] Wilkinson, M. D., Dumontier, M., Aalbersberg, I. J., Appleton, G., Axton, M., Baak, A., Blomberg, N., Boiten, J., da Silva Santos, L. B., Bourne, P. E., Bouwman, J., Brookes, A. J., Clark, T., Crosas, M., Dillo, I., Dumon, O., Edmunds, S., Evelo, C. T., Finkers, R., Gonzalez-Beltran, A., Gray, A. J. G., Groth, P., Goble, C., Grethe, J. S., Heringa, J., ’t Hoen, P. A. C., Hooft, R., Kuhn, T., Kok, R., Kok, J., Lusher, S. J., Martone, M. E., Mons, A., Packer, A. L., Persson, B., Rocca-Serra, P., Roos, M., van Schaik, R., Sansone, S., Schultes, E., Sengstag, T., Slater, T., Strawn, G., Swertz, M. A., Thompson, M., van der Lei, J., van Mulligen, E., Velterop, J., Waagmeester, A., Wittenburg, P., Wolstencroft, K., Zhao, J. & Mons, B. (2016). *Sci. Data*, **3**, 160018.

[bb34] Willett, P., Cole, J. C. & Bruno, I. J. (2020). *CrystEngComm*, **22**, 7233–7241.

[bb35] wwPDB (2023). *Distribution of Electron Microscopy metadata in PDBx/mmCIF file format.* https://www.wwpdb.org/news/news#651c49e2d78e004e766a96a4.

